# Stage-Specific Transcriptome and Proteome Analyses of the Filarial Parasite *Onchocerca volvulus* and Its *Wolbachia* Endosymbiont

**DOI:** 10.1128/mBio.02028-16

**Published:** 2016-11-23

**Authors:** Sasisekhar Bennuru, James A. Cotton, Jose M. C. Ribeiro, Alexandra Grote, Bhavana Harsha, Nancy Holroyd, Amruta Mhashilkar, Douglas M. Molina, Arlo Z. Randall, Adam D. Shandling, Thomas R. Unnasch, Elodie Ghedin, Matthew Berriman, Sara Lustigman, Thomas B. Nutman

**Affiliations:** a Laboratory of Parasitic Diseases, NIAID, NIH, Bethesda, Maryland, USA; b Wellcome Trust Sanger Institute, Wellcome Genome Campus, Hinxton, Cambridgeshire, United Kingdom; c Laboratory of Malaria and Vector Research, NIAID, NIH, Rockville, Maryland, USA; d Department of Biology, Center for Genomics and Systems Biology, New York University, New York, New York, USA; e University of South Florida, Tampa, Florida, USA; f Antigen Discovery Inc., Irvine, California, USA; g College of Global Public Health, New York University, New York, New York, USA; h New York Blood Center, New York, New York, USA

## Abstract

Onchocerciasis (river blindness) is a neglected tropical disease that has been successfully targeted by mass drug treatment programs in the Americas and small parts of Africa. Achieving the long-term goal of elimination of onchocerciasis, however, requires additional tools, including drugs, vaccines, and biomarkers of infection. Here, we describe the transcriptome and proteome profiles of the major vector and the human host stages (L1, L2, L3, molting L3, L4, adult male, and adult female) of *Onchocerca volvulus* along with the proteome of each parasitic stage and of its *Wolbachia* endosymbiont (*w*Ov). In so doing, we have identified stage-specific pathways important to the parasite’s adaptation to its human host during its early development. Further, we generated a protein array that, when screened with well-characterized human samples, identified novel diagnostic biomarkers of *O. volvulus* infection and new potential vaccine candidates. This immunomic approach not only demonstrates the power of this postgenomic discovery platform but also provides additional tools for onchocerciasis control programs.

## IMPORTANCE

The global onchocerciasis (river blindness) elimination program will have to rely on the development of new tools (drugs, vaccines, biomarkers) to achieve its goals by 2025. As an adjunct to the completed genomic sequencing of *O. volvulus*, we used a comprehensive proteomic and transcriptomic profiling strategy to gain a comprehensive understanding of both the vector-derived and human host-derived parasite stages. In so doing, we have identified proteins and pathways that enable novel drug targeting studies and the discovery of novel vaccine candidates, as well as useful biomarkers of active infection.

## INTRODUCTION

Onchocerciasis or river blindness, caused by infection with *Onchocerca volvulus*, is a neglected tropical disease (NTD) that is associated with significant morbidity and disability in the 17 million people currently estimated to be infected (1). Onchocerciasis was the first NTD targeted for control in 1974 by the World Health Organization and is now one of the six NTDs targeted for elimination (2). Currently, the strategy for elimination of *O. volvulus* focuses on controlling transmission through ivermectin-based mass drug administration (MDA) programs that have largely eliminated onchocerciasis in the Americas (3) and that have made significant progress toward that goal in some regions of Africa (4). However, according to recent reports, onchocerciasis cannot be eliminated through MDA with ivermectin solely (5) and may require an estimated 1.30 billion ivermectin treatments until 2045 (6). In addition, ivermectin is contraindicated in areas of coendemicity with *Loa loa*, where the risk of severe (occasionally fatal) adverse events is associated with high levels of circulating *L. loa* microfilariae (mf) (7). Furthermore, the potential for ivermectin resistance (8), the lack of macrofilaricidal activity by ivermectin, and the long time line (>20 years) needed for transmission interruption (5, 6) have prompted research into the development of new tools (macrofilaricidal drugs, diagnostics, and vaccines), the basis of which relies on a fundamental understanding of the parasite’s biology.

Humans are the only definitive hosts of *O. volvulus*. Because there are no existing small-animal models for propagating the life cycle (see Fig. S1 in the supplemental material) of *O. volvulus*, approaches that require parasite material from most stages have been difficult. For example, adult parasites must be obtained surgically from subcutaneous nodules, the mf from human skin, and the infective larvae must be obtained from infected blackflies—a process that, to date, requires feeding of newly hatched naive blackflies on infected humans with microfiladermia. Nevertheless, using relatively limited parasite material from most of the life cycle stages, we have comprehensively profiled the stage-specific transcriptomes of *O. volvulus*, as well as the proteomes of both *O. volvulus* and its *Wolbachia* endosymbiont (*w*Ov), a process made possible by having a high-quality assembly and annotation of the entire *O. volvulus* and *w*Ov genomes (9). Systematic comparisons across the parasite stages and across related nematodes have offered insights into the unique biology of this long-lived filarial parasite, while an immunomic approach has leveraged this information to allow the identification of potential vaccine and diagnostic candidates.

## RESULTS

### Transcriptome and proteome of *O. volvulus.*

Transcriptome data obtained using RNA-seq obtained from most of the vector- and human-derived stages (see Fig. S1 in the supplemental material) of the parasite were not only used to help curate the *O. volvulus* genome annotation (9) but were used in the present study to understand stage-specific mRNA expression. Over 75% of the genes had 100% transcript coverage in all of the stages, with the exception of the adult female worm (http://exon.niaid.nih.gov/transcriptome/O_volvulus/v245/Ov-v245-web.xlsx), most of which, in contrast to the adult male, is composed of uterine tissue containing all of the intrauterine stages that range from early embryos through pretzel-like immature mf to mature mf (10). Because many transcripts with expression levels of <1 RPKM (reads per kilobase of transcript per million mapped reads) were found to encode proteins whose products were validated in separate proteomic analyses, they also have been included in the analyses (https://exon.niaid.nih.gov/transcriptome/O_volvulus/Additional_file_1.xlsx).

**FIG 1:**
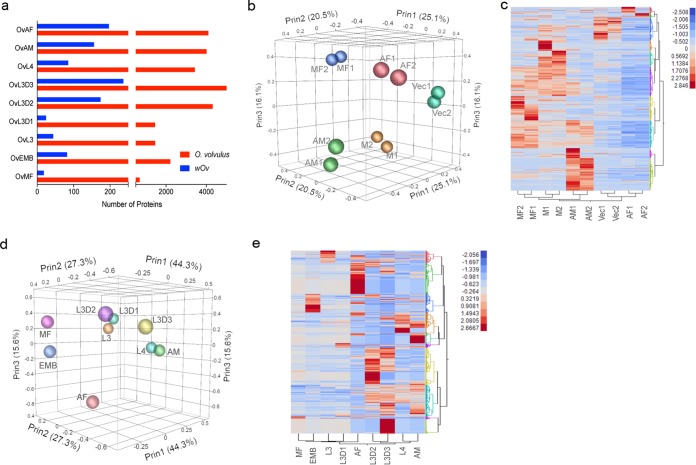
Overview of the transcriptome and proteome of *O. volvulus*. (a) Numbers of *O. volvulus*-specific (red) and *Wolbachia*-specific (blue) proteins identified across the stages by proteomic analyses. (b) Three-dimensional principal-component analysis plots highlighting the transcriptomic signatures of adult males (AM), adult females (AF), mf (MF), vector stages (Vec), and molting (M). (c) Heat map illustrating the hierarchical clustering of the normalized expression of transcripts of the stages in panel b. (d) Three-dimensional principal-component analysis plot highlighting the stage-specific proteomic signatures in adult females (AF), adult males (AM), embryos (EMB), mf, the L2 larval stage, the L3 larval stage, L3 larvae cultured *in vitro* for 1 to 3 days (L3D1, L3D2, and L3D3), and *in vitro*-developed L4 larvae (e). Heat map illustrating the hierarchical clustering of the normalized protein abundance in the stages analyzed in panel d.

The median coverage over the length of any predicted protein by peptides derived from the proteome of each of the stages profiled ranged between ~10 and 15% (see Fig. S2 in the supplemental material). A total of 7,774 *O. volvulus* proteins were identified across all of the stages, resulting in the validation of >64% of the predicted proteins (9). There was maximal proteomic coverage during the development of L3 to L4; adult male and female worms had the second most extensive coverage (Fig. 1a). We were also able to identify/validate the presence 465 of the 785 *Wolbachia* (*w*Ov) proteins predicted by the genomic sequencing of *w*Ov (Fig. 1a and the entire *w*Ov data set at http://exon.niaid.nih.gov/transcriptome/O_volvulus/v245/wOv_web/wOv_Web.xlsx). Overall, there was a high degree of correlation (*P* < 0.0001) between the RPKM (for the transcriptome) and the normalized protein abundance (for the proteome) (see Fig. S2 in the supplemental material), with *r* values that ranged from 0.25 to 0.39, values considered acceptable for global comparisons (11, 12).

**FIG 2:**
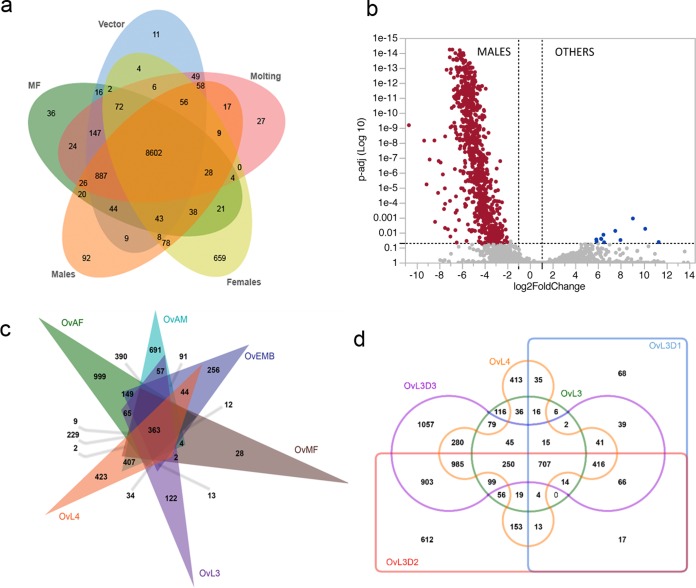
Stage specificity of *O. volvulus* transcriptomes and proteomes. (a) Venn diagram illustrating the numbers of stage-specific transcripts identified in mf, vector-derived stages, molting larvae, adult males, and adult females. (b) Genes differentially expressed in adult males compared to all other stages plotted using log_2_-fold changes on the *x* axis and adjusted *P* values (log_10_) on the *y* axis. (c, d) Venn diagrams illustrating the numbers of stage-specific proteins identified by mass spectrometry in the major somatic stages (c) and during the L3-to-L4 molt (d).

Multivariate analysis using closely related/overlapping larval stages as replicates (*r* = >0.9; see Fig. S3 in the supplemental material) revealed stage-specific transcript profiles that clearly segregated the L2 and L3 vector-derived stages (Vec1, Vec2), the early human developmental stages mimicked *in vitro* (L3 to L4 molting; M1, M2), microfilarial stages (MF1, MF2), adult males (AM1, AM2), and adult females (AF1, AF2) (Fig. 1b and c). Proteomic analyses (Fig. 1d and e) indicate a bias toward male-like expression profiles as the parasites develop from L3 to L4, suggesting a slight selective advantage for the males during early development. As expected from the principal-component analyses, there was a clear stage-specific clustering of the transcriptional and proteomic expression profiles (Fig. 1c and e). Although relatively few transcripts were expressed only in a single stage, except for the females (Fig. 2a), the adult males appeared to have the highest number of differentially expressed transcripts (Fig. 2b). Comparative analysis based on *Caenorhabditis elegans* germline expression data (13) indicated that a majority (69%) of the male-associated differential gene expression is related to spermatogenesis (see Fig. S3 in the supplemental material and https://exon.niaid.nih.gov/transcriptome/O_volvulus/Additional_file_2.xlsx). Inferences, however, from *C. elegans* also appear to classify 6% of the *O. volvulus* male-enriched transcripts as oogenic. Of note, the mf have significantly increased expression of two hypothetical proteins (OVOC1851 and OVOC1852) that are unique to *O. volvulus*, along with OVOC1611, a member of the serine protease inhibitor (SPI) family (see Fig. S3 in the supplemental material).

Amino acid repeats make up 12 to 14% of most of the proteomes studied to date (14). A total of 1,590 *O. volvulus* proteins were found to harbor single amino acid repeats, tandem repeats, or sequence repeat regions (https://exon.niaid.nih.gov/transcriptome/O_volvulus/Additional_file_3.xlsx). In addition to the transcription factors and signaling proteins, uncharacterized proteins had the highest number of amino acid repeats in *O. volvulus* (https://exon.niaid.nih.gov/transcriptome/O_volvulus/Additional_file_4.tif). Proteins containing polyglutamine repeats are common in most organisms, including *O. volvulus* (https://exon.niaid.nih.gov/transcriptome/O_volvulus/Additional_file_4.tif) and other nematodes. While their exact role in* O. volvulus* is not clear, the expression of polyglutamine repeats was shown to be neurotoxic in both *C. elegans* and *Drosophila* and is dynamically regulated throughout the lifetime of an organism, suggesting a link to longevity (15). Interestingly, one of the lead *O. volvulus* vaccine candidates (16), Ov-RAL-2 (OVOC9988), has 11 glutamine residues in its amino terminus. These residues are probably not important for the protective ability of this protein, as the *Brugia malayi* homologue (Bm2001), that is also protective in animals, does not have this repeat (17).

### Stage-specific functional enrichment.

Although many of the putative proteins were classified into functional categories, ~20% were considered to be uncharacterized and unique (or divergent) (9). Clustering of these unique genes on the basis of transcript and protein abundances indicates not only that distinct subsets are enriched at specific stages (https://exon.niaid.nih.gov/transcriptome/O_volvulus/Additional_file_5.tif) but also that 7% have signatures (SignalP and SecretomeP 2.0) indicative of secretion (secreted-divergent) (see the *w*Ov data set at http://exon.niaid.nih.gov/transcriptome/O_volvulus/v245/wOv_web/wOv_Web.xlsx). Although the functions of these uncharacterized gene products remain largely unknown, identifying the stages in which they are highly expressed may provide a method to infer their functions on the basis of associations with particular developmental processes, such as molting or embryogenesis.

There were no significant differences in the total number of genes or proteins identified per functional category across the various stages (https://exon.niaid.nih.gov/transcriptome/O_volvulus/Additional_file_6.tif). However, genes involved in metabolism, cytoskeletal elements, protein modification, protein export, proteasome, and transcription machinery had significantly lower expression (adjusted *P* < 0.001, analysis of variance [ANOVA]) in adult females than in all of the other stages. Gene set enrichment analyses (GSEA) indicated that selected functional categories were enriched on the basis of the stage (see Fig. S4 in the supplemental material). The adult female stage was associated with pathways linked to detoxification and the extracellular matrix (see Fig. S4). This enriched subset of extracellular-matrix-related genes was primarily composed of collagens and gene products associated with chitin synthesis machinery. Although the mf are an integral part of the fertile adult female, genes corresponding to NADH dehydrogenase activity (gene ontology [GO] category 0008137) and cytochrome *c* oxidase activity (GO category 0004129) were enriched in the adult females compared to all other stages. In contrast to the adult female worms, the mf stages showed significant enrichment of genes associated with protein synthesis (ribosomal proteins) and protein modification, with cyclophilins and chaperones (heat shock proteins) as the major contributors. These are likely part of the machinery required for cellular morphogenesis that occurs after the parasite is ingested by the blackfly vector. The expression of genes representing nuclear regulation was enriched in adult males, an enrichment that could be attributed to DNA replication and spermatogenesis. In addition, molecular functions of nucleotide binding (GO category 0000166), peptidase activity (GO category 0070011), and phosphoprotein phosphatase activity (GO category 0004721) were the top GO processes enriched in adult male *O. volvulus*.

### Insights into development.

Analyses of protein abundance in each of the stages identified 363 proteins expressed in all of the somatic stages (Fig. 2c). Genes encoding proteins involved in metabolism, cytoskeletal elements, and protein modification made up more than 50% of these core genes. Proteins common in OvEMB and OvAF are likely to play a role in oogenesis and/or embryogenesis. Similarly, proteins identified exclusively during the L3-to-L4 molt highlight the machinery required during this important first developmental molt after adaption to the human host environment (Fig. 2d). On the basis of *C. elegans* RNA interference data, *O. volvulus* homologues of *C. elegans* that are associated with embryonic lethality (EMB), larval arrest (LVA), larval lethality (LVL), defective molting (MLT), and lethality (LET) phenotypes were observed to be enriched not only in the embryos, mf (and thereby adult females), and L3 larval stages (https://exon.niaid.nih.gov/transcriptome/O_volvulus/Additional_file_7.tif) but also in adult males. Similarly, *O. volvulus* encodes orthologues of the most critical genes essential for molting (on the basis of *C. elegans*; https://exon.niaid.nih.gov/transcriptome/O_volvulus/Additional_file_8.xlsx) during the L3-to-L4 transition.

The analyses also highlight other proteins that have been shown to be essential for molting and/or other developmental processes of filarial parasites. For example, embryogenesis and molting of filarial parasites are dependent on the activity of cathepsin L-like cysteine proteases (CPLs). During molting, CPLs stored in the glandular esophagus are released and help in the breakdown of the old cuticle and possibly support the synthesis of a new cuticle (18, 19). Analyses using EnsemblCompara (20) indicate an expansion of CPL-like enzymes in *O. volvulus* (9). Significantly increased expression of CPL and CPZ molecules (21) was observed in L2 and L3 larvae compared to that in other stages (*P* < 0.002, ANOVA). While their role during L3-to-L4 molting is known (19, 22, 23), their increased expression in L2 larvae suggests that CPLs and CPZ also play a role in the L2-to-L3 molt in the vector, similar to what was described in *B. malayi* (24). Interestingly, the GO categories of GTPase activity (GO category 0003924), oxidoreductase activity (GO category 0016491), procollagen-proline dioxygenase activity (GO categories 0004656 and 0019798), peptidyl-proline dioxygenase activity (GO categories 0031543 and 0031545), and protein disulfide oxidoreductase activity (GO category 0015035) were among the categories represented by the differentially expressed genes during both the L2-to-L3 and L3-to-L4 molting events. GSEA also identified an immunologically important class of molecules as being enriched in L3 larval stages (see Fig. S4 in the supplemental material) and a set of extracellular-matrix-related genes distinct from those extracellular matrix proteins enriched in adult female worms. Some of these include collagens that can be regulated by prolyl-4 hydroxylases, part of an expanded family of genes based on the analysis of the *O. volvulus* genome (9) that also shows differential expression across the various stages (https://exon.niaid.nih.gov/transcriptome/O_volvulus/Additional_file_9.tif).

### Gene families—protein kinases, G protein-coupled receptors (GPCRs), and nuclear hormone receptors (NHRs).

Approximately 55% of the predicted proteins had significant matches (<1E-05) to Pfam databases, with a protein kinase domain (Pkinase, PF00069), an RNA recognition motif (RRM_1, PF00076), and a seven-transmembrane receptor (7TM_1, PF00001) being the top three represented categories. The Pkinase domain forms the catalytic center of protein kinases that are involved in a wide variety of cellular processes influencing apoptosis, differentiation, and embryonic development. Their increased stage-specific increased expression (see Fig. S5 in the supplemental material) likely reflects their role in germline differentiation (in adult worms) or in larval developmental stages (mf and L3). Comparative analyses of the *O. volvulus* kinome (see Table S1 in the supplemental material) with other filarial and nonfilarial control kinomes resulted in the identification of *O. volvulus* kinases that are unique or belong to nematode-specific families (25) (see Table S2 in the supplemental material). More interestingly, the expression of casein kinase 1 and its subfamily the Tau tubulin kinase-like (CK1/TTBKL) and FER subfamily of tyrosine kinases (TK/FER) was enriched in adult males (https://exon.niaid.nih.gov/transcriptome/O_volvulus/Additional_file_10.xlsx). Both CK1 and Fer kinases have been implicated in mammalian Wnt signaling. The non-receptor tyrosine kinase of the Fer family was also shown to be important in spermatogenesis (26). These nematode-specific kinases—clearly distinct from human, fruit fly, and yeast kinases—may provide insights into comparative worm biology studies and are very tractable drug targets.

**FIG 3:**
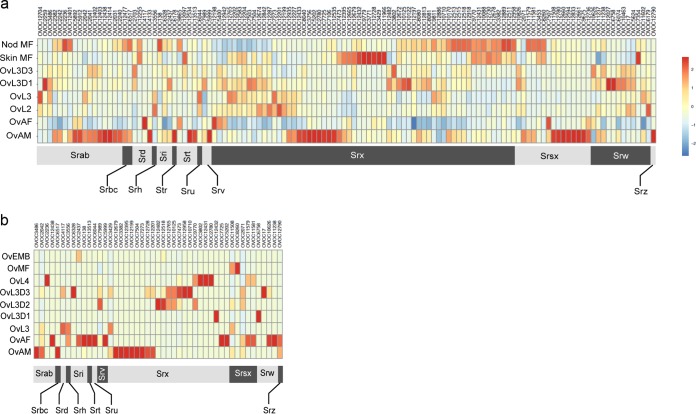
*O. volvulus* stage-specific expression of G protein-coupled receptors (GPCRs). Shown are heat maps depicting the stage-specific expression of GPCRs in *O. volvulus* ordered by family. Values plotted are the *z* scores of RPKM (a) and normalized spectral abundance factors of proteins (b). Red to blue denotes high to low expression, as shown on the scale to the right.

Among the seven-transmembrane receptors, the GPCR gene families are greatly expanded in *O. volvulus* (9). Hierarchical clustering of the transcriptional and proteomic expression data reveals clear patterns of stage-specific expression (Fig. 3). Some members of the Srx family show very high expression in the skin and nodular mf, while other families show increased expression in adult males. These patterns reveal unique chemosensory requirements for certain stages of *O. volvulus*, including migratory adult males and skin mf, compared to the more stationary female worms.

The NHRs are known to play an important role in nematode developmental processes (27). In comparison to *C. elegans* and *B. malayi*, the NHR repertoire is smaller in *O. volvulus* (9) but still contains all five NHR genes that play a role in molting and embryogenesis (28, 29). Indeed, the *O. volvulus* ecdysone receptor (EcR, OVOC9104) and NHR RXR (OVOC2435) show increased expression during the L3-to-L4 molt (https://exon.niaid.nih.gov/transcriptome/O_volvulus/Additional_file_11.tif). Furthermore, GSEA indicate enrichment of other potential NHRs (such as OVOC351 and OVOC353) involved in signal transduction processes of adult female worms (*P* < 0.0001; false discovery rate, <1%). Similarly, orthologues of the *C. elegans* NHRs *nhr-6* (OVOC8200), *nhr-23* (OVOC464), *nhr-25* (OVOC2839), *nhr-41* (OVOC4741), and *nhr-85* (OVOC827), known to be involved in molting and metamorphosis, were detected as transcripts or proteins during the *in vitro* L3-to-L4 molt. In addition, NHRs implicated in neural differentiation (OVOC635, OVOC3708) and sex determination (OVOC5276) had increased expression levels in the molting stages, reflecting their likely role in development. The distinct expression profile of OVOC2265 (*nhr-32*) predominantly in the proteomes of the nodular mf and embryonic stages (https://exon.niaid.nih.gov/transcriptome/O_volvulus/Additional_file_11.tif) suggests a possible role in embryogenesis. Among other embryo-enriched transcripts and proteins, OVOC11613 (immunodominant or major antigen) (30) and OVOC9384 (Oveg1) (31) were shown to be related to embryogenesis as well. Moreover, though the precise role of *Ce*-LFI-1 (orthologue of OVOC11613) in development is not yet known, it interacts with LIN-5, which is essential for proper spindle positioning and chromosome segregation (32), an essential feature of cell division.

### Secretome and host-parasite interactions.

Approximately 20% of the genes in the *O. volvulus* genome are predicted to be secreted through classical secretion mechanisms, and about ~42% are predicted to be secreted through nonclassical mechanisms. All filarial worms are known to release excretory/secretory (E/S) products that are critical components in the helminth arsenal of proteins that perform diverse functions (33). These include (i) modulation of the host immune response, (ii) remodeling of host tissue, (iii) alteration of host tissue nutritional status, and (iv) enhancement of larval tissue migration (34). The *O. volvulus* genome contains many of these immunologically relevant genes (9), among which are the L3-enriched or mf-enriched cystatins that have been shown to interfere with antigen processing and presentation to T cells (35), small SPIs (Ov-SPI-1, Ov-SPI-2), and serpins (https://exon.niaid.nih.gov/transcriptome/O_volvulus/Additional_file_12.tif). SPIs also play an important role in controlling the molting process (36) and immune evasion (37). The analysis of the *O. volvulus* genome revealed the presence of 10 additional SPIs beyond the 2 previously identified, Ov-SPI-1 and Ov-SPI-2 (9). Eleven of these 12 are highly expressed during the L3-to-L4 molt, consistent with their potential role in molting or immune invasion during the first days of establishment in the human host (https://exon.niaid.nih.gov/transcriptome/O_volvulus/Additional_file_12.tif). Interestingly, 2 of the 12 SPIs are highly expressed in adult males, consistent with a previous study demonstrating SPIs being localized in sperm within the adult male testis (36). The other immunologically relevant protein enrichment was the *O. volvulus* adult male-enriched expression of indoleamine 2,3-dioxygenase, an interferon-inducible enzyme that suppresses adaptive T-cell immunity, and the enrichment of the homologue of suppressor of cytokine signaling 7 (SOCS7; OVOC678) in L3/L4 developmental stages.

Stage-specific expression of serine, aspartic, cysteine, and metallo-proteases reflects their roles in host invasion, molting and migration in nematodes (https://exon.niaid.nih.gov/transcriptome/O_volvulus/Additional_file_13.tif). Scanning of the *O. volvulus* genome, notably, identified the presence of 13 chitinases, 8 of which belong to glycosyl hydrolase family 18. We know that the larva-specific chitinase (Ov-CHI-1) plays an important role in parasite transmission, molting, and remodeling of the L4 cuticle (38), the inhibition of which inhibits larval molting (38, 39). The relatively higher expression of Ov-CHI-1 in the L2 larval stages suggests a probable role in migration through insect tissues (https://exon.niaid.nih.gov/transcriptome/O_volvulus/Additional_file_13.tif). The increased expression of what had been thought to be larva-specific chitinases in the *O. volvulus* adult male—seen in both transcriptomic and proteome data—raises the possibility that these glycosyl hydrolase 18 family molecules have another functional role not previously recognized.

### *Wolbachia* proteome.

**FIG 4:**
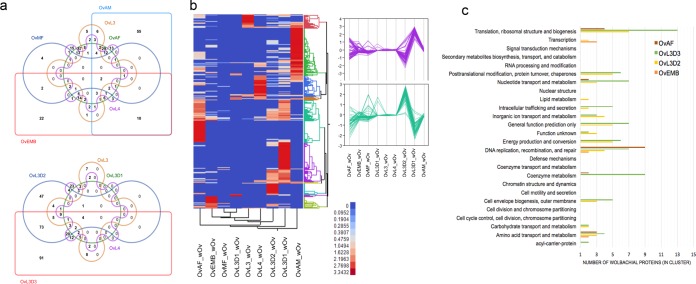
The *w*Ov proteome. (a) Shown are Venn diagrams illustrating the number of stage-specific *w*Ov proteins identified in the somatic proteomes of the major life stages (top) and during the L3-to-L4 molt (bottom). (b) Heat map of *w*Ov stage-specific protein expression with blue to red indicating low to high expression and parallel coordinate plots of protein clusters specifically expressed during the L3-to-L4 molt (right). (c) Number of *w*OV proteins with increased expression during the L3-to-L4 molt (OvL3D2, OvL3D3), in adult females (OvAF), and in embryos (OvEMB) by functional category.

*Wolbachia*, a genus of *Alphaproteobacteria*, infects an estimated two-thirds of all arthropod species. The members of this genus are probably the most abundantly and vertically transmitted organisms that exist in facultative or obligate endosymbiotic associations (40). In nematodes, their prevalence is restricted to two families of worms, *Onchocercidae* and *Pratylenchidae* (41), with *Onchocercidae* exhibiting widespread obligate mutualism, as evidenced by retarded larval growth (42), embryostasis (43), and death (44) when the *Wolbachia* bacteria are eliminated. Because we performed RNA-seq analyses with poly(A)-enriched transcripts, *w*Ov transcript identification was limited. Proteomic analyses, however, identified 465 *w*Ov proteins, some of which were common to or uniquely expressed in specific stages of *O. volvulus* (Fig. 1a and Fig. 4a). Comparative analyses with the *Wolbachia* symbiont of the bovine parasite *Onchocerca ochengi* (*w*Oo) (45) was limited because of a lack of corresponding stage-specific protein identifications. However, there was a significant correlation with the number of peptides of wolbachial origin identified from adult *O. ochengi* females (OoAF) and OvAF and OvEMB (see Fig. S6 in the supplemental material). Similar to what was observed in the proteomes of *w*Bm (46) and *w*Oo (45), the GroEL (wOv00466), outer membrane protein (wOv00621), outer surface protein (WSP, wOv00566), and molecular chaperone DnaK (wOv00687) were among the most commonly identified abundant proteins (top 25) across most of the stages. Clustering of the normalized protein abundance values resulted in the identification of *w*Ov clusters that were specifically OvAF, OvEMB, OvL3D2, or OvL3D3 abundant (Fig. 4b). Among the clusters overrepresented in OvL3-to-L4 development (Fig. 4b, right), many could be mapped to functional categories, with the top five functions being (i) translation, ribosomal structure, and biogenesis; (ii) posttranslational modification, protein turnover, and chaperone; (iii) energy production and conversion; (iv) coenzyme metabolism and cell envelope biogenesis, and (v) outer membrane proteins (Fig. 4c).

The increase in proteins related to particular functions in certain developmental stages may reflect multiplication of *Wolbachia* bacteria within these defined stages. In these stages, such as the developing L3 and in the adult female (and thus during embryogenesis), when the metabolic needs are the greatest, it is possible that the increased *w*Ov replication seen may reflect the symbiotic needs of the *O. volvulus* parasite during growth and development (47).

### Biomarkers—an immunomic approach.

**FIG 5:**
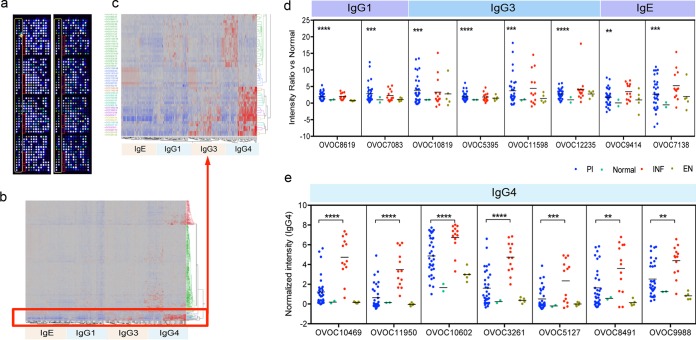
*O. volvulus* protein array. (a) Quality control of *O. volvulus* protein array chips with anti-HA (right) and anti-His (left) antibodies. (b) Heat map depicting the clustered data from all samples for all isotypes with a blowup of the red-boxed image in panel c highlighting the cluster of IgG4-reactive proteins. Red to blue denotes high to low reactivity. (d) Scatter plots of representative proteins with significant IgG1, IgG3, and IgE responses (potentially allergenic) in putatively immune individuals compared to infected individuals, plotted as ratios with respect to normal serum samples. **, *P* ≤ 0.01; ***, *P* ≤ 0.001; ****, *P* < 0.0001 (one-way ANOVA corrected for multiple comparisons [Holm-Sidak]). (e) Scatter plot of representative proteins with significant IgG4 reactivity in infected individuals, plotted as normalized intensity. PI, putatively immune; INF, infected individuals; EN, endemic normal individuals; Normal, pools of healthy normal donors.

Using an immunomic approach (48–50), we profiled host antibody responses to a subset of parasite stage-specific proteins (397 proteins). These selected proteins (https://exon.niaid.nih.gov/transcriptome/O_volvulus/Additional_file_14.xlsx) were printed on a protein array, quality checked, and assessed for isotype-specific antibody responses (IgG1, IgG3, IgG4, and IgE) (Fig. 5b and c) by using serum samples from 52 individuals comprising *O. volvulus*-infected, putatively immune, and control individuals from Ecuador (51), Guatemala (52), and Cameroon (53). After normalization, clusters specific for IgG4, IgG3 and/or IgG1, and IgE reactivities were identified. Further analyses led to the identification of OVOC10819, OVOC5395, OVOC11598, OVOC12235, OVOC8619, and OVOC7083 as potential novel vaccine candidates (Fig. 5d) on the basis of significant IgG1 and/or IgG3 reactivity (with little to no IgE reactivity [54]) or potentially allergenic proteins that induce elevated levels of IgE (OVOC9414, OVOC7138). The identification of proteins that are highly expressed by the mf (see Table S3 in the supplemental material and https://exon.niaid.nih.gov/transcriptome/O_volvulus/Additional_file_15.tif) and are specifically recognized by serum samples from protected individuals opens up new possibilities for the development of a safe antitransmission or therapeutic vaccine (55). Because IgG4 reactivity to filarial antigens provides better serodiagnostic species specificity (56, 57) than other isotypes, the data were also analyzed for IgG4-reactive proteins. On the basis of the IgG4 responses (Fig. 5e), we were able to identify heretofore unrecognized biomarkers of active infection (e.g., OVOC10469, OVOC10602, OVOC11950, OVOC3261, OVOC5127, OVOC8491, and OVOC9988). Indeed, we have successfully tested and validated OVOC10469 in a luciferase immunoprecipitation system (LIPS) immunoassay that highlights not only the utility of this protein as a biomarker of active *O. volvulus* infection (https://exon.niaid.nih.gov/transcriptome/O_volvulus/Additional_file_16.tif) but also the usefulness of this type of immunomic approach.

## DISCUSSION

Using a combination of transcriptomic and proteomic data, we performed stage-specific analyses that are, to our knowledge, the most comprehensive for any of the filarial parasites. Because of the limited availability of *O. volvulus* larval-stage-specific material, we used the transcriptomes of the closely related/overlapping larval stages as biological replicates, since their expression patterns were significantly similar (see Fig. S3 in the supplemental material and https://exon.niaid.nih.gov/transcriptome/O_volvulus/Additional_file_17.xlsx).

Further, for the proteomic analyses, we chose depth of coverage over distribution of the limited stage-specific sample for technical replicates, with the rationale that although a difference in transcript (RNA) and protein recovery from the various stages is expected, normalized data (RPKM and spectral abundance) provide provisional evidence of the relative abundance of any particular gene or protein at a given stage. This data set thus provides an in-depth resource for understanding and analyzing the biological pathways that are critical for the development of the various stages of the parasite in the vector and human hosts, in host-worm interactions, and for the identification of novel biomarkers (e.g., OVOC10469, OVOC3261, and OVOC12838) and targets for interventions. Comparative stage-specific proteomic analyses of the recently available *O. ochengi* (58) proteome and the corresponding *O. volvulus* stages demonstrate a relatively high degree of correlation (https://exon.niaid.nih.gov/transcriptome/O_volvulus/Additional_file_18.tif), albeit a weaker one than that with the more distant species *B. malayi* (46) (https://exon.niaid.nih.gov/transcriptome/O_volvulus/Additional_file_19.tif) that is likely due to differences in coverage depth (58).

Natural immunity to *O. volvulus* can be acquired in affected populations by a few individuals who have been qualified as being putatively immune (52, 59, 60). The hypothesis is that they are protected because they are able to mount an appropriate immune response against the L3 larvae; surface proteins of L3 larvae and/or the E/S products released by molting larvae are considered to be an important source of such protective antigens (59). The identification of *O. volvulus* unique proteins that are larva-specific or the identification of adult and/or mf stage-specific biomarkers suggests that tools are already available to help achieve the lofty goal of onchocerciasis elimination in the coming decades.

## MATERIALS AND METHODS

Detailed materials and methods are available in Text S1 in the supplemental material.

### Parasite samples.

Parasite material used for RNA-seq and proteomic analyses was collected at the research facility at the Tropical Research Station, Kumba, Cameroon (53), or in Ecuador (51). Adult worm samples were obtained from nodules excised during nodulectomies. L3 larvae were obtained from *Simulium damnosum* flies 7 to 8 days after infection with skin mf as described previously (61). Fresh L3 larvae were also cultured *in vitro* at 37°C for 1 to 3 days (L3D1, L3D2, and L3D3) or for 6 days when they molt and L4 were then collected. Nodular and skin mf were purified as described previously (62, 63). Embryonic stages were purified from mf and eggs that were extruded into the medium during the process of cleaning female adult worms.

### Human serum samples.

All human serum samples were obtained by using protocols approved by the Institutional Review Board of either the National Institute of Allergy and Infectious Diseases (Guatemala or Ecuador) or by the New York Blood Center and the Tropical Research Station, Kumba, Cameroon. All infected people were microfilaria positive on the basis of skin snip analyses.

### RNA-seq, assembly, and analyses.

RNA-seq libraries were prepared from parasite stages in accordance with the RNA-seq protocols of the Illumina mRNA-Seq Sample Prep kit and the Illumina TruSeq kit. The reads were analyzed as previously described (64). Genes that had blast scores of <30% of the maximum possible score (self-blast) in other nematodes with E values of >1E-05 were considered unique. The normalized transcriptome data were analyzed in JMP Genomics (SAS Inc., Cary, NC) for general assessment of distribution analyses, correlations, principal-component analyses, ANOVA, hierarchical clustering and heat map generation, and parallel coordinate plots.

### Protein isolation.

For proteomic analyses, additional stages of embryos (OvEMB) and L3D2 (OvL3D2) and L4 (OvL4) larvae were also analyzed. Total soluble proteins were extracted from all of the stages, dialyzed, desalted, and digested with trypsin.

### Liquid chromatography-tandem mass spectrometry.

Liquid chromatography was performed with the Easy-nLC 1000 UHPLC system (Thermo). The spectra were searched by using a combined database of *O. volvulus*, *w*Ov, and human proteins. Proteins were required to have one or more unique peptides across the samples analyzed with E values of ≤0.0001.

### Protein arrays.

The genes of interest were cloned into T7 expression vector pXT7. Proteins were expressed with a coupled *in vitro* transcription and translation system, the *Escherichia coli*-based cell-free Rapid Translation System 100 High Yield kit (5 Prime), as 10×His-tagged or hemagglutinin (HA)-tagged fusion proteins and printed onto arrays. The arrays were probed with serum samples, and isotype reactivity was detected with Cy5-labeled antibodies. The slides were scanned, and reactivity was quantified. The quantified data were normalized and analyzed for significant proteins.

### LIPS assay.

For evaluation of antibody titers, a standard antibody-based LIPS assay (65) was performed.

### Accession number(s).

The entire *O. volvulus* data set analyzed is available as a hyperlinked Excel workbook at http://exon.niaid.nih.gov/transcriptome/O_volvulus/v245/Ov-v245-web.xlsx; the *w*Ov data set is available as a hyperlinked Excel workbook at http://exon.niaid.nih.gov/transcriptome/O_volvulus/v245/wOv_web/wOv_Web.xlsx. The RNA-seq data are available under BioProject PRJEB2965 (*O. volvulus*) under the accession numbers shown in the supplemental material. The mass spectrometry proteomic data have been deposited in the ProteomeXchange Consortium via the PRIDE partner repository under the data set identifier PXD003585.

## SUPPLEMENTAL MATERIAL

Text S1  Supplemental methods used in this study. Download Text S1, DOCX file, 0.1 MB

Figure S1  Life cycle of *O. volvulus* sampled. Illustrated are the various stages of *O. volvulus* analyzed by RNA-seq and/or mass spectrometry. L2 larval stages (OvL2) and infective L3 larvae were obtained from infected blackflies (L3 larvae, OvL3). L3 larvae were cultured *in vitro* for 1 (OvL3D1), 2 (OvL3D2), or 3 (OvL3D3) days. L4 larvae (OvL4) were obtained after molting. Adult male (OvAM) and adult female (OvAF) worms were obtained from nodules. Mf were obtained from nodules (Nodular MF, NodMF) or skin (SknMF). Download Figure S1, TIF file, 12.5 MB

Figure S2  *O. volvulus* stage-specific proteome coverage and correlations of transcriptomes and proteomes across all available stages. (a) Box plot with outliers representing the distribution of peak area intensity (log_10_) of proteins identified across all stages of *O. volvulus*. (b) Box plot with outliers showing the overall 10 to 15% coverage of all of the proteins by spectra identified by mass spectrometry across all stages. (c to h) Plotted on a log-log scale are the transcriptome abundance (RPKM) on the *y* axis and the normalized spectral abundance of the proteome on the *x* axis for OvAF (c), OvAM (d), OvMF (e), OvL3 (f), OvL3D1 (g), and OvL3D3 (h). The *r* and *P* values of the Spearman rank correlation are shown in red on each plot. Download Figure S2, TIF file, 14.6 MB

Figure S3  Correlation between replicates and closely related/overlapping stages and differentially expressed genes. Plotted on the *x* and *y* axes are the log-transformed transcriptome abundances (RPKM) of nodular and skin mf (a), vector-derived L2 and L3 larvae (b), L3 larvae cultured for 1 and 3 days (during molting) (c), biological replicates of adult males (d), and biological replicates of adult females (e). The *r* value of the Spearman rank correlation is shown on each plot. (f) Pie chart showing that the distribution of the majority (69%) of adult male enriched genes (with significant homology to *C. elegans*) map to spermatogenic processes. (g, h) Volcano plots illustrating the differentially expressed genes between adult males (blue) and adult females (red) (g) and between mf (red) and the rest of the stages (h). Plotted on the *x* axis are the log 2-fold changes, and plotted on the *y* axis are the adjusted *P* values. Interesting and unique genes are identified. Download Figure S3, TIF file, 17.7 MB

Figure S4  GSEA. Illustrated is the stage-specific enrichment of gene sets involved in specific functional categories in *O. volvulus* adults (left), in mf (middle), and during L3-L4 development (right). GSEA of transcripts ranked by their relative abundance in each stage was performed. The green curve shows the enrichment score and reflects the degree to which each protein (represented by the vertical lines) is represented at the top or bottom of the ranked list. The heat maps below the curves depict the relative abundances (red to blue indicates high to low expression) of the transcripts specifically enriched in a specific stage in comparison with other stages in the corresponding functional classes. Download Figure S4, TIF file, 10.9 MB

Figure S5  *O. volvulus* stage-specific expression of protein kinases. The heat map shown depicts the stage-specific expression (log_2_-transformed RPKM) of Pkinase (PF00069) domain-containing proteins. Red to blue denotes high to low expression, as shown on the right. Download Figure S5, TIF file, 9.8 MB

Figure S6  Comparative analysis of *w*Ov proteins. (a) The correlation between the number of peptides identified in *Wolbachia* bacteria derived from adult females of *O. volvulus* (*w*Ov; *w*OvAF) and in *Wolbachia* bacteria from adult females of *O. ochengi* (*w*Oo; *w*OoAF). (b) The relationship between the numbers of peptides identified in *Wolbachia* bacteria derived from embryos of *O. volvulus* (*w*Ov; *w*OvEMB) and in *Wolbachia* bacteria derived from adult females of *O. ochengi* (*w*Oo; *w*OoAF). (c) The Venn diagram illustrates the four most abundant *wOv* proteins detected across all of the stages. Download Figure S6, TIF file, 13 MB

Table S1  The *O. volvulus* kinome. The classification of *O. volvulus* kinases into families and subfamilies is shown. Table S1, XLSX file, 0.1 MB

Table S2  Filarial kinomes. Comparative analyses of the kinomes of the major filarial parasites and control organisms are shown. Table S2, XLSX file, 0.1 MB

Table S3  Stage-specific proteins and their corresponding transcriptomic profiles. Table S3, XLSX file, 0.2 MB
