# Supplementary material for: Stage-Specific Transcriptome and Proteome Analyses of the Filarial Parasite Onchocerca volvulus and Its Wolbachia Endosymbiont
Source: mBio. 2016 Nov 23;7(6):e02028-16. doi: 10.1128/mBio.02028-16 (PMC5137501; doi:10.1128/mBio.02028-16)
Supplement: Text S1 — Supplemental methods used in this study. Download [file mbo006163100s1.docx]

**Detailed Methods**

**Parasite Samples**

Parasite material used for RNAseq and proteomic analyses were collected at the research facility at the Tropical Research Station, Kumba, Cameroon (1); or in Ecuador (2). Adult worm samples were obtained from nodules excised during nodulectomies. The nodules were treated with collagenase as described previously (3) or with slight modifications to that protocol. Briefly, individual and cleaned freshly obtained nodules were immersed in 0.5% collagenase (Sigma grade IV) in RPMI 1640 containing 10% FCS supplemented with 200 units of penicillin and 200 µg/ml streptomycin. The flat tubes containing the nodule were then placed in a rocking water bath and incubated at 35**°**C until the tissue was digested completely. Alternatively, frozen nodules were thawed, cleaned and digested with LiberaseTL (Roche) in Hanks Balanced Salt Solution (HBSS) supplemented with 3 mM CaCl_2_. When digested, the liberated adult worms were unraveled from residual tissue with mounted needles under a dissecting scope, and then washed in several changes of RPMI 1640 or HBSS. The cleaned adult worms were stored at -80**°**C until use.

L3 larvae were produced at the Tropical Medicine Research Station, Kumba, Cameroon. They were obtained from *Simulium damnosum* flies 7–8 days after infection with skin microfilariae as described previously (4). After dissection and washing, the larvae were cryopreserved (5) and shipped to the USA. Fresh L3 larvae were also cultured *in vitro* in groups of 10 larvae in 96-well plates containing a 1:1 mixture of Iscove’s modified Dulbecco medium and NCTC-135, 20% FCS and antibiotic-antimycotic (Life Technologies, Gaithersburg, MD) for 3-days at 37°C. Larvae were collected after 1, 2 or 3 days in culture, washed with Tris-EDTA and snap frozen in liquid nitrogen. Nodular and skin microfilariae were purified as described previously (6, 7). Embryonic stages were purified from mf and eggs that are extruded into the medium during the cleaning process. The medium was collected and centrifuged at 1000 rpm for 10 mins at room temp. The pellet containing the mix of microfilariae and embryonic stages was resuspended and layered on LSM (MP Biomedicals, CA) and centrifuged at 500rpm for 15 mins with the brake off. The purified embryonic stages that form the pellet were washed and stored at -80°C until use.

**Human Serum Samples**

All human serum samples were obtained using protocols approved by the IRBs of either the National Institute of Allergy or Infectious Disease (Guatemala or Ecuador) or by the New York Blood Center and the Tropical Research Station, Kumba (Cameroon). Written consent was obtained from all subjects. For those unable to read or write, a thumbprint was made in the presence of a literate witness whose signature was also required. All infected people were microfilaria positive on skin snip. Those termed putatively immune met the criteria set forth previously (8). None of the samples came from subjects who had received prior treatment ivermectin.

**Transcriptome Sequencing, Assembly and Analyses**

High-throughput transcriptome data were generated from the RNA of *O. volvulus* stages: nodular microfilariae (NodMF), skin microfilariae (SknMF), L2 (OvL2), L3 (OvL3), L3 day 1 (OvL3D1), L3 day 3 (OvL3D3), adult male (OvAM) and adult female worms (OvAF), as part of the *O. volvulus* genome (9). The RNA and protein lysates were made from pools of 3000-5000 L3 larval stages, 2-5 adult worms. For all larval stages and adult worms, RNA was prepared using TRIzol and lysing matrix D (1.4-mm ceramic spheres) and a Fastprep24 (MP Biomedicals). RNAseq libraries were prepared following the RNAseq protocols of the Illumina mRNA-Seq Sample Prep kit and the Illumina TruSeq kit. Transcriptome libraries were sequenced on Illumina HiSeq 2000 or MiSeq machines. The reads were analyzed as previously described(10), and reproduced here with small changes. Reads were trimmed of low quality regions (<13), and those with an average quality of 20 or more were used. Illumina primers were removed from the sequences following a parallel blastn of the reads against HiSeq TruSeq adapters. Resulting reads were assembled with ABySS software(11) using various kmer (k) values (every fifth from 21 to 91). The reads were mapped to the predicted gene set(9) using blastn(12) with a word size of 25, masking homonucleotide decamers and allowing mapping to up to three different CDS if the BLAST results had the same score. Using an in-house pipeline(13) automated annotation of proteins was based on a vocabulary of nearly 290 words found in matches to various databases, including Swissprot, Gene Ontology, KOG, Pfam, and SMART, Refseq-invertebrates and a subset of the GenBank sequences containing nematode protein sequences, as well as the presence or not of signal peptides and transmembrane domains. Protein repeats were analyzed using repseq(14) and reptile ([www.reptile.unibe.ch](http://www.reptile.unibe.ch)) algorithms. Signal peptide, transmembrane domains, furin cleavage sites, and mucin-type glycosylation were determined with software from the Center for Biological Sequence Analysis (Technical University of Denmark, Lyngby, Denmark)(15-17). Classification of *O. volvulus* and other filarial kinases was done by Kinannote(18). Genes that had blast scores <30% of max possible score (self blast) in other nematodes with an e-value greater than 1E-05 were considered as ‘unique’. To be *O. volvulus* unique, the genes were also compared with the genomes of *O. flexuosa* and *O. ochengi.* Further manual annotation was done as required. The analyses data was mapped into a hyperlinked Excel spreadsheet as previously described(19).

Transcriptome data (normalized to Reads Per Kilobase of transcripts per Million - RPKM) from the excel spreadsheet were imported into JMP Genomics (SAS Inc., Cary NC) for general assessment of distribution analyses, correlations, principal component analyses, analysis of variation (ANOVA), hierarchical clustering and heatmap generation, and parallel co-ordinate plots. Heatmaps of clustering analyses were also done in R using an array of packages. Differential expressing of genes was analyzed using DESeq(20). Because of the paucity of biological material, and the significant correlations between the transcriptomes of closely related/overlapping stages (**Figure S4**), nodular microfilariae and skin microfilariae were used as replicates for ‘microfilaria’; OvL2 and OvL3 stages as replicates for ‘vector’ derived and OvL3D1 and OvL3D3 as replicates for ‘molting’. Gene Ontology (GO) enrichment analyses were done using topGO in R package, and mapped using QuickGO(21). GSEA analyses were as previously described(19). GSEA examines the clustering of the pre-defined group of genes (gene set) across the entire database to identify biased gene expression in specific stages. Briefly, the entire list of *O. volvulus* transcriptome or proteome was sorted on their relative abundances (RPKM or normalized spectral abundance factors). The distribution of proteins from an a priori defined set throughout the ranked list was then determined by GSEA(22).

**Protein Depletion, Denaturation, Digestion and Desalting**

For proteomic analyses, additional stages of embryos (OvEMB), L3D2 (OvL3D2) and L4 larvae (OvL4) were also analyzed. Total soluble proteins from all the stages were extracted using the UPX universal protein extraction kit (Protein Discovery, San Diego) as per manufacturer’s instructions and quantified using Pierce BCA assay (ThermoFisher Scientific). Extracted protein samples (20-40 ug) were prepared for digestion using the filter-assisted sample preparation (FASP) method(23). Briefly, the samples were suspended in 1% SDC, 50 mM Tris-HCl, pH 7.6, 3 mM DTT, sonicated briefly, and incubated in a Thermo-Mixer at 40°C, 1000 RPM for 20 min. Samples were centrifuged to clarify and the supernatant was transferred to a 3.0 kD MWCO device (Millipore) and centrifuged at 13000 x g for 30 min. The sample was buffer exchanged with 1% SDC, 100 mM Tris-HCl, pH 7.6, then alkylated with 15 mM iodoacetamide. The SDC concentration was reduced to 0.1%. Samples were digested overnight using trypsin at an enzyme to substrate ratio of 1:100, at 37°C in a Thermo-Mixer at 1000 RPM. Digested peptides were collected by centrifugation. Twenty micrograms of the digested peptides, were desalted using reversed phase stop-and-go extraction (STAGE) tips(24). Peptides were eluted with 80% acetonitrile, 0.2% trifluoroacetic acid and lyophilized in a SpeedVac (Thermo Savant) to near dryness, approximately 1 h.

**Liquid Chromatography-Tandem Mass Spectrometry**

Each digestion mixture was analyzed by UHPLC-MS/MS. LC was performed on the Easy-nLC 1000 UHPLC system (Thermo). Mobile phase A was 97.5% MilliQ water, 2% acetonitrile, 0.5% acetic acid. Mobile phase B was 99.5% acetonitrile, 0.5% acetic acid. The 240 min LC gradient ran from 0% B to 35% B over 210 min, then to 80% B for the remaining 30 min. Samples were loaded directly to the column. The column was 50 cm x 75 um I.D. and packed with 2 micron C18 media (Thermo Easy Spray PepMap). The LC was interfaced to a quadrupole-Orbitrap mass spectrometer (Q-Exactive, Thermo) via nano-electrospray ionization using a source with an integrated column heater (Thermo Easy Spray source). The column was heated to 50°C. An electrospray voltage of 2.2 kV was applied. The mass spectrometer was programmed to acquire, by data-dependent acquisition, tandem mass spectra from the top 20 ions in the full scan from 400-1200 m/z. Dynamic exclusion was set to 15s, singly-charged ions were excluded, isolation width was set to 1.6 Da, full MS resolution to 70,000 and MS/MS resolution to 17,500. Normalized collision energy was set to 25, automatic gain control to 2e5, max fill MS to 20 ms, max fill MS/MS to 60 ms and the underfill ratio to 0.1%.

**Data Processing and Library Searching**

Mass spectrometer RAW data files were converted to mzML format using msconvert(25). MGF files were generated from mzML using the Peak Picker HiRes tool, part of the OpenMS framework(26). All searches were performed on Amazon Web Services-based cluster compute instances using the Proteome Cluster interface. Detailed search parameters are printed in the search output XML files. Briefly, all searches required 10 ppm precursor mass tolerance, 0.02 Da fragment mass tolerance, strict tryptic cleavage, up to 2 missed cleavages, fixed modification of cysteine alkylation, variable modification of methionine oxidation and protein expectation value scores of 0.0001 or lower. Proteome Cluster builds species- and genus-specific protein sequence libraries monthly from the most current UniProtKB distribution(27). MGF files were searched using the most recent protein sequence libraries available from UniProtKB or using the FASTA libraries of *O. volvulus* (WS245) and its endosymbiont *Wolbachia* (*wOv*). MGF files were searched using X!!Tandem(28) using both the native(29) and k-score(30) scoring algorithms and by OMSSA(31). XML output files were parsed and non-redundant protein sets determined using Proteome Cluster based on previously published rules(32). MS1-based isotopic features were detected and peptide peak areas were calculated using the FeatureFinderCentroid tool, part of the OpenMS framework(26). Proteins were required to have 1 or more unique peptides across the analyzed samples with E-value scores of 0.0001 or less. Abundance of proteins is measured by peak area intensity and by normalized spectral abundance factor (NSAF)(33), where $\mathrm{NSAF}=\frac{{(\frac{\mathrm{Spectra}}{\mathrm{Length}})}_{p}}{\sum_{p=1}^{n} {(\frac{\mathrm{Spectra}}{\mathrm{length}})}_{p}}$. The mass spectrometry proteomics data have been deposited to the ProteomeXchange Consortium via the PRIDE partner repository with the dataset identifier PXD003585.

**Protein Array**

**Array Construction and Probing**. The following cDNA libraries from OvAM (**SAW98MLW-OvAM),** OvAF (**SAW98MLW-OvAF)**, OvL2 (**SAW98MLW-OvL2)**, OvL3 (**SAW94WL-OvL3)**, molting L3 (**SL96MLW-OvML3**), MF (**SAW98MLW-OvM**F) were obtained from the NIH/NIAID Filariasis Research Reagent Resource Center ([www.filariasiscenter.org](http://www.filariasiscenter.org)) and used to amplify selected gene products. Molting larval transcripts that were not amplified successfully from the cDNA libraries were subsequently obtained from oligo dT cDNA prepared from RNA purified from OvL3D1, OvL3D2 or OvL3D3 (SuperScript® III First-Strand Synthesis System, Invitrogen). *In vivo* recombination cloning was performed as described previously(34). Briefly, PCR primers were designed as 40 mer oligonucleotides with 20 sequence specific bases and a 20-base adapter sequence. The adapter sequences were designed to be homologous to the cloning site of the linearized T7 expression vector pXT7 and allow the PCR products to be cloned by homologous recombination in *E. coli* DH5α cells. PCR reactions were set up using Hot Master Mix (5 Prime) plus DMSO (5%). The recommended cycling conditions were used and PCR products were checked for correct size using an agarose gel. PCR products were mixed with linearized pXT7 vector and were transformed into DH5α competent cells. DNA was purified using Qiaprep 96 Turbo Miniprep Kit (Qiagen). Resulting clones were checked for insert on an agarose gel and were sent for sequencing (Retrogen).

**Chip Fabrication**.

Proteins were expressed using a coupled *in vitro* transcription and translation (IVTT) system, *E. coli* based cell-free Rapid Translation System (RTS) 100 High Yield Kit (5 Prime), from the *O. volvulus* expressible clone library following the manufacturer’s manual with the exception of adding detergent to the IVTT master mix at a final concentration of 0.1% Brij 78. Shortlisted *O. volvulus* proteins were synthesized using IVTT in disulfide-bond folded formats and printed onto an array. Known immunogenic proteins (purified recombinant proteins) were also printed as positive controls.

Approximately 1 nL of unpurified IVTT reactions were spotted onto 8-pad nitrocellulose coated Oncyte Avid Slides (GraceBio Labs) using an OmniGrid Accent microarray printer (Digilab) equipped with a 946 Printhead and 946MP4 Spotting Pins (ArrayIt). Each IVTT expressed protein includes an N-terminal 10X polyhistidine (HIS) epitope tag and C-terminal hemagglutinin (HA) epitope tag. Microarray chip printing and protein expression were quality checked by probing random slides with mouse anti-polyHIS (Sigma), rat anti-HA (Roche) and rabbit anti-*E. coli* (LifeSpan BioSciences). Antibodies were diluted 1:1,000 in a 3 mg/mL *E. coli* DH5α lysate solution in protein arraying buffer (GVS Filter Technology) and incubated at room temperature for 30 min. Chips, FAST Slide Holders (GVS Filter Technology) and FAST Slide Incubation Chambers (GVS Filter Technology) were assembled and nitrocellulose pads were hydrated using 100 µL blocking buffer for 30 min at room temperature with rocking. Blocking buffer was removed, pre-incubated antibodies were added and chips were incubated for two hours at room temperature, washed three times with 1X TBS-0.05% Tween 20, followed by incubation with Cy5-conjugated goat anti-mouse IgG Fcɣ, Cy5-conjugated goat anti-rat IgG Fcɣ or Cy5-conjugated goat anti-rabbit IgG Fcɣ (Jackson ImmunoResearch) diluted 1:400 in blocking buffer for one hour at room temperature with agitation. Chips were washed three times with 1X TBS-0.05% Tween 20, three times with 1X TBS, and once with water. Chips were air dried by centrifugation at 500 x g for 10 min, stored in a light proof desiccator for at least 2 hours and scanned on a GenePix 4300 with Autoloader (Molecular Devices) using the 635 nm laser. Resulting 16-bit TIFF images were quantified using GenePix Pro Microarray Analysis Software (Molecular Devices) and a GenePix Array List (GAL) file. Spot and background intensities were measured and median spot values minus local background (M635-B) values were exported as comma delimited file (CSV).

**Probing Samples**. Serum samples were diluted 1:200 in a 3 mg/mL *E. coli* DH5α lysate solution in protein arraying buffer and incubated at room temperature for 30 min. Chips, FAST Slide Holders and FAST Slide Incubation Chambers were assembled and nitrocellulose pads were hydrated using 100 µL blocking buffer for 30 min at room temperature with rocking. Blocking buffer was removed, pre-incubated serum samples were added and chips were incubated overnight at 4**°**C with agitation. The following day chips were washed three times with 1X TBS-0.05% Tween 20, followed by incubation with biotin-conjugated anti-human secondary antibodies against IgG1, IgG3, IgG4 or IgE (SigmaAldrich) diluted 1:1,000 in blocking buffer for one hour at room temperature with agitation. Chips were washed three times with 1XTBS-0.05% Tween 20, followed by incubation with streptavidin-conjugated SureLight P-3 (Columbia Biosciences) at room temperature protected from light with agitation. Chips were washed three times with 1X TBS-0.05% Tween 20, three times with 1X TBS, and once with water. Chips were air dried by centrifugation at 500 x g for 10 min, stored in a light proof desiccator for at least 2 hours and scanned on a GenePix 4300 with Autoloader using the 635 nm laser. Resulting 16-bit TIFF images were quantified using GenePix Pro Microarray Analysis Software and a GAL file. M635-B values were exported for each slide as CSV files.

**Data Analysis**. The individual CSV files were compiled and organized using R into the “raw” data files. The raw data were normalized by dividing the IVTT protein spot intensity by the sample specific median of the IVTT control spots printed throughout the chip, then taking the base-2 logarithm of the ratio. The normalized data provides a relative measure of the specific antibody binding to the non-specific antibody binding to the IVTT controls. Normalized data was imported into JMP Genomics (SAS) and analyzed for antigen reactivity and significance (ANOVA, tested for multiple comparisons using Holm-Sidak method) between the clinical groups and isotypes, and adjusted for multiple comparisons. Significant proteins were graphed in Prism 6.0 (GraphPad).

**Luciferase ImmunoPrecipitation System Assay**

Fusion proteins were made for the selected proteins by cloning the full-length gene into a FLAG-epitope-tagged mammalian *Renilla reniformis* luciferase (Ruc)-containing expression vector, pREN2(35). Extracts (lysates) containing the light-emitting Ruc-antigen fusions were prepared in from transfected 293-F cells (Life Technologies) as previously described(35, 36) and frozen until use. For evaluation of antibody titers, a standard LIPS antibody-based assay was used(36) with slight modifications. Briefly, 100 μl of the assay master mix (20 mM Tris [pH 7.5], 150 mM NaCl, 5 mM MgCl_2_, 1% Triton X-100), 1 μl of undiluted plasma/serum and 1 x 10^6^ light units (LU) of the Ruc-antigen fusion protein were added to each well of a 96-well polypropylene plate. This plate was incubated for 10 minutes at room temperature. 100ul of 1:20 diluted Ultralink protein A/G beads (50% suspension) in phosphate-buffered saline (PBS) was added to 96-well high throughput screening filter plate (Millipore, Bedford MA), and applied to vacuum manifold to retain the Protein A/G beads. The 100 μl antigen-antibody reaction mixture from each microtiter well of the 96-well polypropylene plate was transferred to the filter plate and incubated for 10-15 minutes at room temperature. The filter plate containing the reaction mixture and beads was subjected to vacuum manifold. The retained protein A/G beads were washed with the assay master mix and final wash with PBS. The LU were measured in a Berthold LB 960 Centro microplate luminometer, using a coelenterazine substrate (Promega, Madison WI).

**REFERENCES**

1. **MacDonald AJ, Turaga PS, Harmon-Brown C, Tierney TJ, Bennett KE, McCarthy MC, Simonek SC, Enyong PA, Moukatte DW, Lustigman S.** 2002. Differential cytokine and antibody responses to adult and larval stages of Onchocerca volvulus consistent with the development of concomitant immunity. Infect Immun **70:**2796-2804.

2. **Elson LH, Guderian RH, Araujo E, Bradley JE, Days A, Nutman TB.** 1994. Immunity to onchocerciasis: identification of a putatively immune population in a hyperendemic area of Ecuador. J Infect Dis **169:**588-594.

3. **Schulz-Key H, Albiez EJ, Buttner DW.** 1977. Isolation of living adult Onchocerca volvulus from nodules. Tropenmed Parasitol **28:**428-430.

4. **Lustigman S, Huima T, Brotman B, Miller K, Prince AM.** 1990. Onchocerca volvulus: biochemical and morphological characteristics of the surface of third- and fourth-stage larvae. Exp Parasitol **71:**489-495.

5. **Schiller EL, Turner VM, Figueroa Marroquin H, D'Antonio R.** 1979. The cryopreservation and in vitro cultivation of larval Onchocerca volvulus. Am J Trop Med Hyg **28:**997-1009.

6. **Taylor DW, Goddard JM, McMahon JE.** 1984. Isolation and purification of microfilariae from nodules of Onchocerca volvulus. Trans R Soc Trop Med Hyg **78:**707-708.

7. **Medina-De la Garza CE, Brattig NW, Tischendorf FW.** 1987. Rapid method for the purification of viable microfilariae from nodules of Onchocerca volvulus by Percoll gradient centrifugation. Trop Med Parasitol **38:**53-54.

8. **Ward DJ, Nutman TB, Zea-Flores G, Portocarrero C, Lujan A, Ottesen EA.** 1988. Onchocerciasis and immunity in humans: enhanced T cell responsiveness to parasite antigen in putatively immune individuals. J Infect Dis **157:**536-543.

9. **Cotton JA, Bennuru S, Grote A, Harsha B, Tracey A, Beech R, Doyle SR, Dunn M, Hotopp JCD, Holroyd N, Kikuchi T, Lambert O, Mhashilkar AS, Mutowo P, Nursimulu N, Ribeiro JM, Rogers MB, Stanley E, Swapna LS, Tsai IJ, Unnasch TR, Voronin D, Parkinson J, Nutman TB, Ghedin E, Berriman M, Lustigman S.** 2016. The genome of Onchocerca volvulus, agent of river blindness. Nat Microbiol **In press**.

10. **Ribeiro JM, Schwarz A, Francischetti IM.** 2015. A Deep Insight Into the Sialotranscriptome of the Chagas Disease Vector, Panstrongylus megistus (Hemiptera: Heteroptera). J Med Entomol **52:**351-358.

11. **Simpson JT, Wong K, Jackman SD, Schein JE, Jones SJ, Birol I.** 2009. ABySS: a parallel assembler for short read sequence data. Genome Res **19:**1117-1123.

12. **Altschul SF, Madden TL, Schaffer AA, Zhang J, Zhang Z, Miller W, Lipman DJ.** 1997. Gapped BLAST and PSI-BLAST: a new generation of protein database search programs. Nucleic Acids Res **25:**3389-3402.

13. **Karim S, Singh P, Ribeiro JM.** 2011. A deep insight into the sialotranscriptome of the gulf coast tick, Amblyomma maculatum. PLoS One **6:**e28525.

14. **Depledge DP, Lower RP, Smith DF.** 2007. RepSeq--a database of amino acid repeats present in lower eukaryotic pathogens. BMC Bioinformatics **8:**122.

15. **Duckert P, Brunak S, Blom N.** 2004. Prediction of proprotein convertase cleavage sites. Protein Eng Des Sel **17:**107-112.

16. **Julenius K, Molgaard A, Gupta R, Brunak S.** 2005. Prediction, conservation analysis, and structural characterization of mammalian mucin-type O-glycosylation sites. Glycobiology **15:**153-164.

17. **Sonnhammer EL, von Heijne G, Krogh A.** 1998. A hidden Markov model for predicting transmembrane helices in protein sequences. Proc Int Conf Intell Syst Mol Biol **6:**175-182.

18. **Goldberg JM, Griggs AD, Smith JL, Haas BJ, Wortman JR, Zeng Q.** 2013. Kinannote, a computer program to identify and classify members of the eukaryotic protein kinase superfamily. Bioinformatics **29:**2387-2394.

19. **Bennuru S, Meng Z, Ribeiro JM, Semnani RT, Ghedin E, Chan K, Lucas DA, Veenstra TD, Nutman TB.** 2011. Stage-specific proteomic expression patterns of the human filarial parasite Brugia malayi and its endosymbiont Wolbachia. Proc Natl Acad Sci U S A **108:**9649-9654.

20. **Anders S, Huber W.** 2010. Differential expression analysis for sequence count data. Genome Biol **11:**R106.

21. **Binns D, Dimmer E, Huntley R, Barrell D, O'Donovan C, Apweiler R.** 2009. QuickGO: a web-based tool for Gene Ontology searching. Bioinformatics **25:**3045-3046.

22. **Subramanian A, Tamayo P, Mootha VK, Mukherjee S, Ebert BL, Gillette MA, Paulovich A, Pomeroy SL, Golub TR, Lander ES, Mesirov JP.** 2005. Gene set enrichment analysis: a knowledge-based approach for interpreting genome-wide expression profiles. Proc Natl Acad Sci U S A **102:**15545-15550.

23. **Wisniewski JR, Zougman A, Nagaraj N, Mann M.** 2009. Universal sample preparation method for proteome analysis. Nature Methods **6:**359-U360.

24. **Rappsilber J, Mann M, Ishihama Y.** 2007. Protocol for micro-purification, enrichment, pre-fractionation and storage of peptides for proteomics using StageTips. Nat Protoc **2:**1896-1906.

25. **Chambers MC, Maclean B, Burke R, Amodei D, Ruderman DL, Neumann S, Gatto L, Fischer B, Pratt B, Egertson J, Hoff K, Kessner D, Tasman N, Shulman N, Frewen B, Baker TA, Brusniak MY, Paulse C, Creasy D, Flashner L, Kani K, Moulding C, Seymour SL, Nuwaysir LM, Lefebvre B, Kuhlmann F, Roark J, Rainer P, Detlev S, Hemenway T, Huhmer A, Langridge J, Connolly B, Chadick T, Holly K, Eckels J, Deutsch EW, Moritz RL, Katz JE, Agus DB, MacCoss M, Tabb DL, Mallick P.** 2012. A cross-platform toolkit for mass spectrometry and proteomics. Nat Biotechnol **30:**918-920.

26. **Sturm M, Bertsch A, Gropl C, Hildebrandt A, Hussong R, Lange E, Pfeifer N, Schulz-Trieglaff O, Zerck A, Reinert K, Kohlbacher O.** 2008. OpenMS - an open-source software framework for mass spectrometry. BMC Bioinformatics **9:**163.

27. **UniProt C.** 2015. UniProt: a hub for protein information. Nucleic Acids Res **43:**D204-212.

28. **Bjornson RD, Carriero NJ, Colangelo C, Shifman M, Cheung KH, Miller PL, Williams K.** 2008. X!!Tandem, an improved method for running X!tandem in parallel on collections of commodity computers. J Proteome Res **7:**293-299.

29. **Craig R, Beavis RC.** 2004. TANDEM: matching proteins with tandem mass spectra. Bioinformatics **20:**1466-1467.

30. **MacLean B, Eng JK, Beavis RC, McIntosh M.** 2006. General framework for developing and evaluating database scoring algorithms using the TANDEM search engine. Bioinformatics **22:**2830-2832.

31. **Geer LY, Markey SP, Kowalak JA, Wagner L, Xu M, Maynard DM, Yang X, Shi W, Bryant SH.** 2004. Open mass spectrometry search algorithm. J Proteome Res **3:**958-964.

32. **Slotta DJ, McFarland MA, Markey SP.** 2010. MassSieve: panning MS/MS peptide data for proteins. Proteomics **10:**3035-3039.

33. **Liu H, Sadygov RG, Yates JR, 3rd.** 2004. A model for random sampling and estimation of relative protein abundance in shotgun proteomics. Anal Chem **76:**4193-4201.

34. **Davies DH, Liang X, Hernandez JE, Randall A, Hirst S, Mu Y, Romero KM, Nguyen TT, Kalantari-Dehaghi M, Crotty S, Baldi P, Villarreal LP, Felgner PL.** 2005. Profiling the humoral immune response to infection by using proteome microarrays: high-throughput vaccine and diagnostic antigen discovery. Proc Natl Acad Sci U S A **102:**547-552.

35. **Burbelo PD, Goldman R, Mattson TL.** 2005. A simplified immunoprecipitation method for quantitatively measuring antibody responses in clinical sera samples by using mammalian-produced Renilla luciferase-antigen fusion proteins. BMC Biotechnol **5:**22.

36. **Burbelo PD, Ching KH, Klimavicz CM, Iadarola MJ.** 2009. Antibody profiling by Luciferase Immunoprecipitation Systems (LIPS). J Vis Exp doi:10.3791/1549.
