# Supplementary figures and images for: Stage-Specific Transcriptome and Proteome Analyses of the Filarial Parasite Onchocerca volvulus and Its Wolbachia Endosymbiont
Source: mBio. 2016 Nov 23;7(6):e02028-16. doi: 10.1128/mBio.02028-16 (PMC5137501; doi:10.1128/mBio.02028-16)

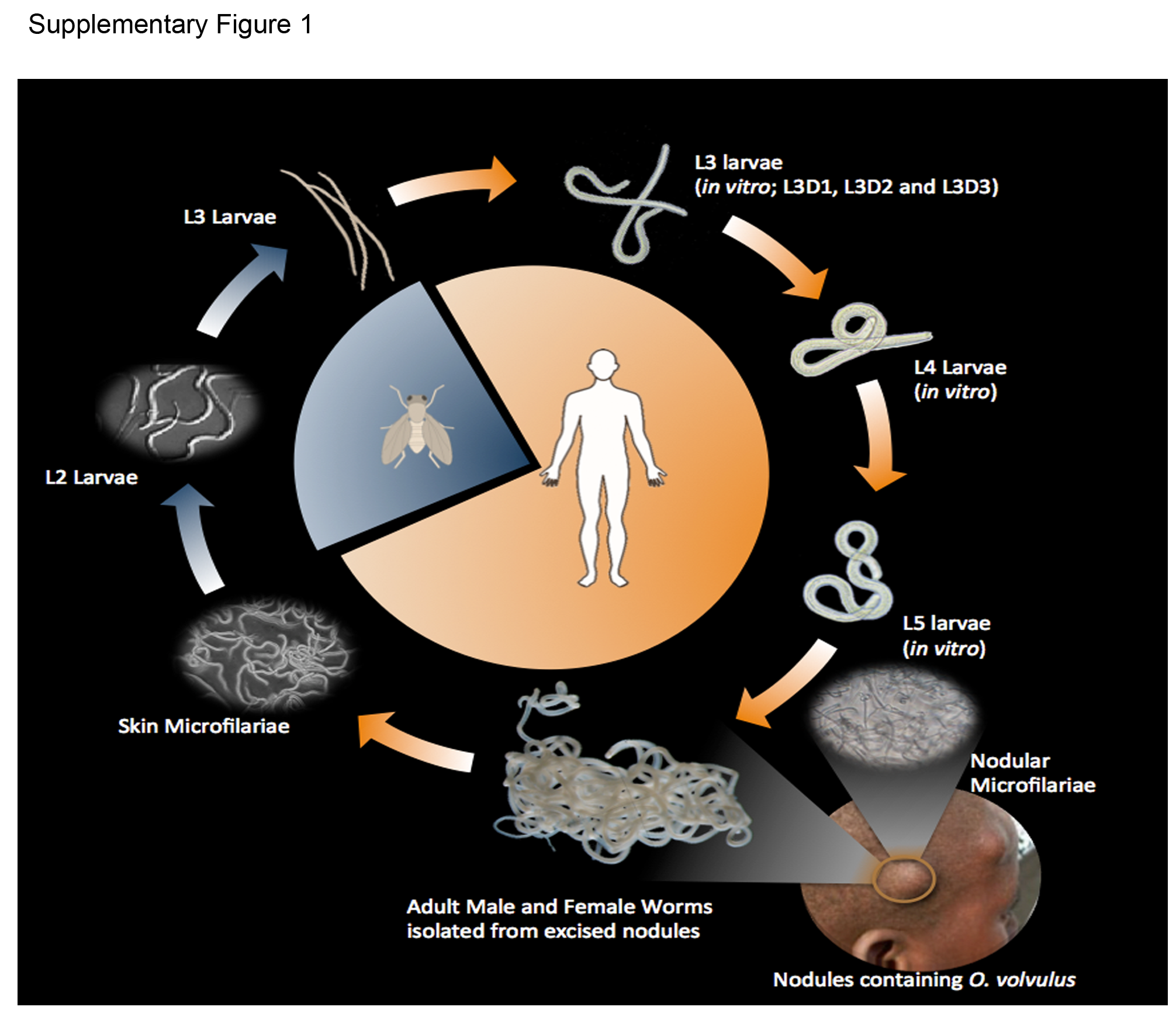

Supplement: Figure S1 — Life cycle of O. volvulus sampled. Illustrated are the various stages of O. volvulus analyzed by RNA-seq and/or mass spectrometry. L2 larval stages (OvL2) and infective L3 larvae were obtained from infected blackflies (L3 larvae, OvL3). L3 larvae were cultured in vitro for 1 (OvL3D1), 2 (OvL3D2), or 3 (OvL3D3) days. L4 larvae (OvL4) were obtained after molting. Adult male (OvAM) and adult female (OvAF) worms were obtained from nodules. Mf were obtained from nodules (Nodular MF, NodMF) or skin (SknMF). Download [file mbo006163100sf1.tif]

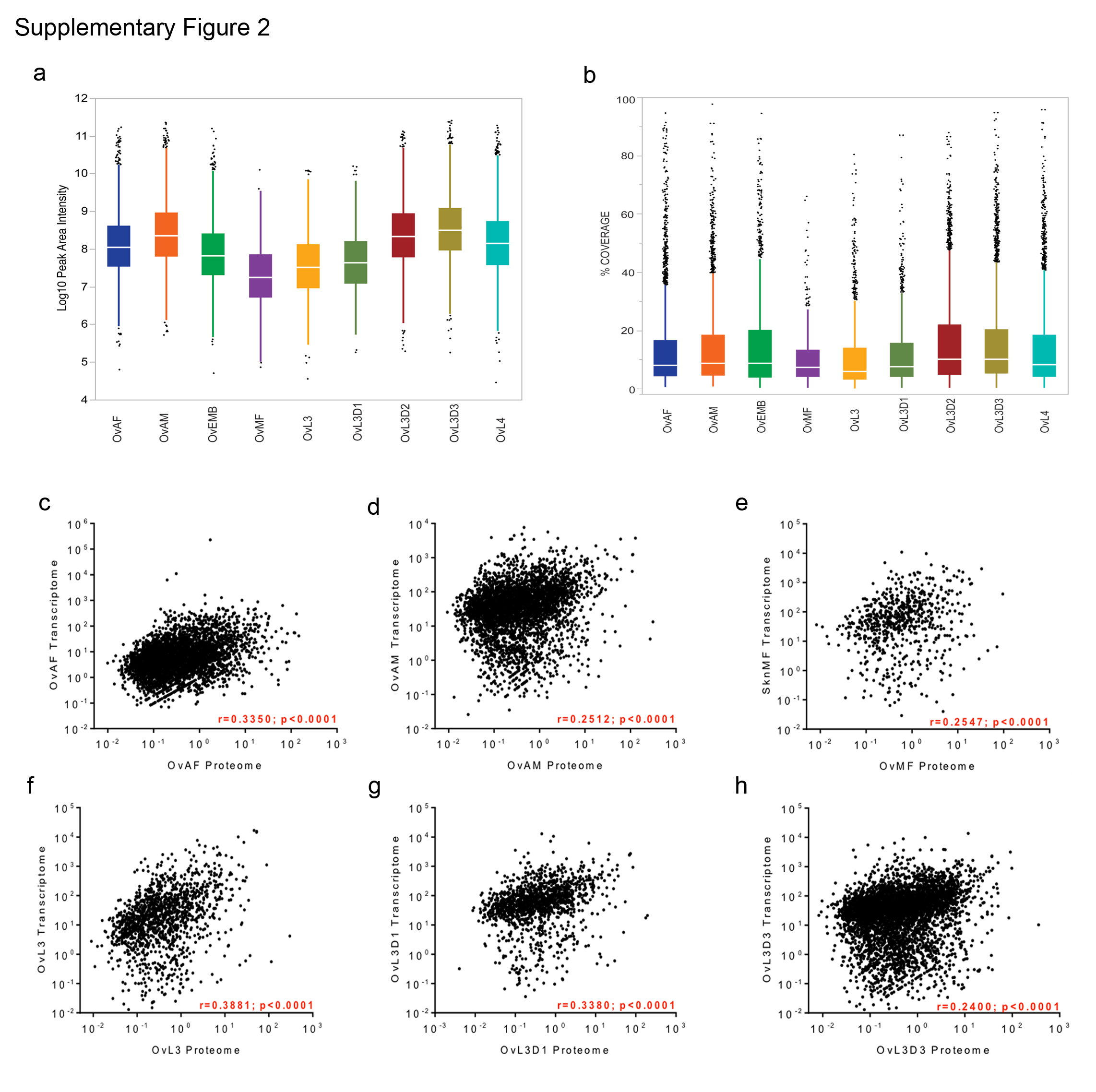

Supplement: Figure S2 — O. volvulus stage-specific proteome coverage and correlations of transcriptomes and proteomes across all available stages. (a) Box plot with outliers representing the distribution of peak area intensity (log10) of proteins identified across all stages of O. volvulus. (b) Box plot with outliers showing the overall 10 to 15% coverage of all of the proteins by spectra identified by mass spectrometry across all stages. (c to h) Plotted on a log-log scale are the transcriptome abundance (RPKM) on the y axis and the normalized spectral abundance of the proteome on the x axis for OvAF (c), OvAM (d), OvMF (e), OvL3 (f), OvL3D1 (g), and OvL3D3 (h). The r and P values of the Spearman rank correlation are shown in red on each plot. Download [file mbo006163100sf2.tif]

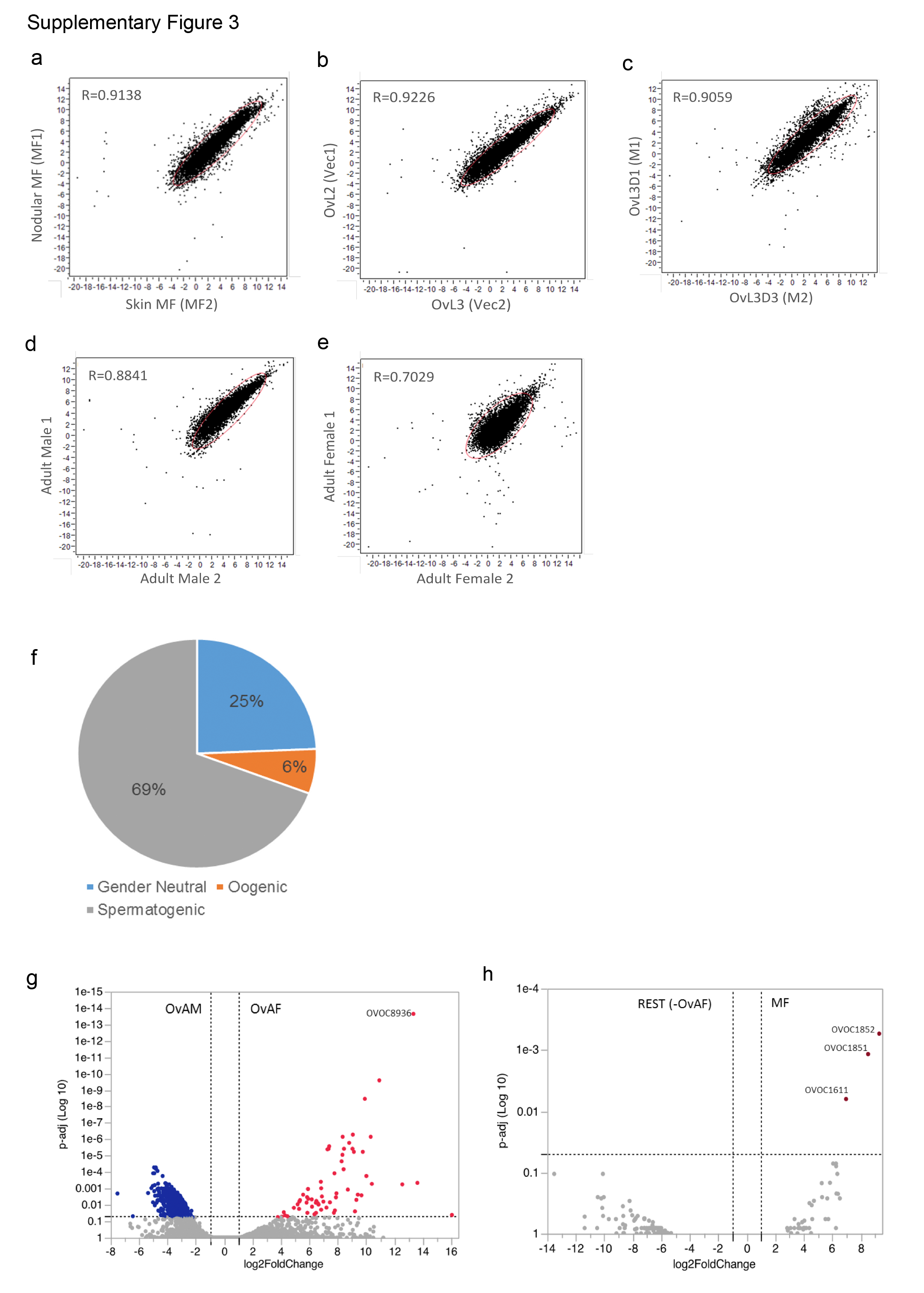

Supplement: Figure S3 — Correlation between replicates and closely related/overlapping stages and differentially expressed genes. Plotted on the x and y axes are the log-transformed transcriptome abundances (RPKM) of nodular and skin mf (a), vector-derived L2 and L3 larvae (b), L3 larvae cultured for 1 and 3 days (during molting) (c), biological replicates of adult males (d), and biological replicates of adult females (e). The r value of the Spearman rank correlation is shown on each plot. (f) Pie chart showing that the distribution of the majority (69%) of adult male enriched genes (with significant homology to C. elegans) map to spermatogenic processes. (g, h) Volcano plots illustrating the differentially expressed genes between adult males (blue) and adult females (red) (g) and between mf (red) and the rest of the stages (h). Plotted on the x axis are the log 2-fold changes, and plotted on the y axis are the adjusted P values. Interesting and unique genes are identified. Download [file mbo006163100sf3.tif]

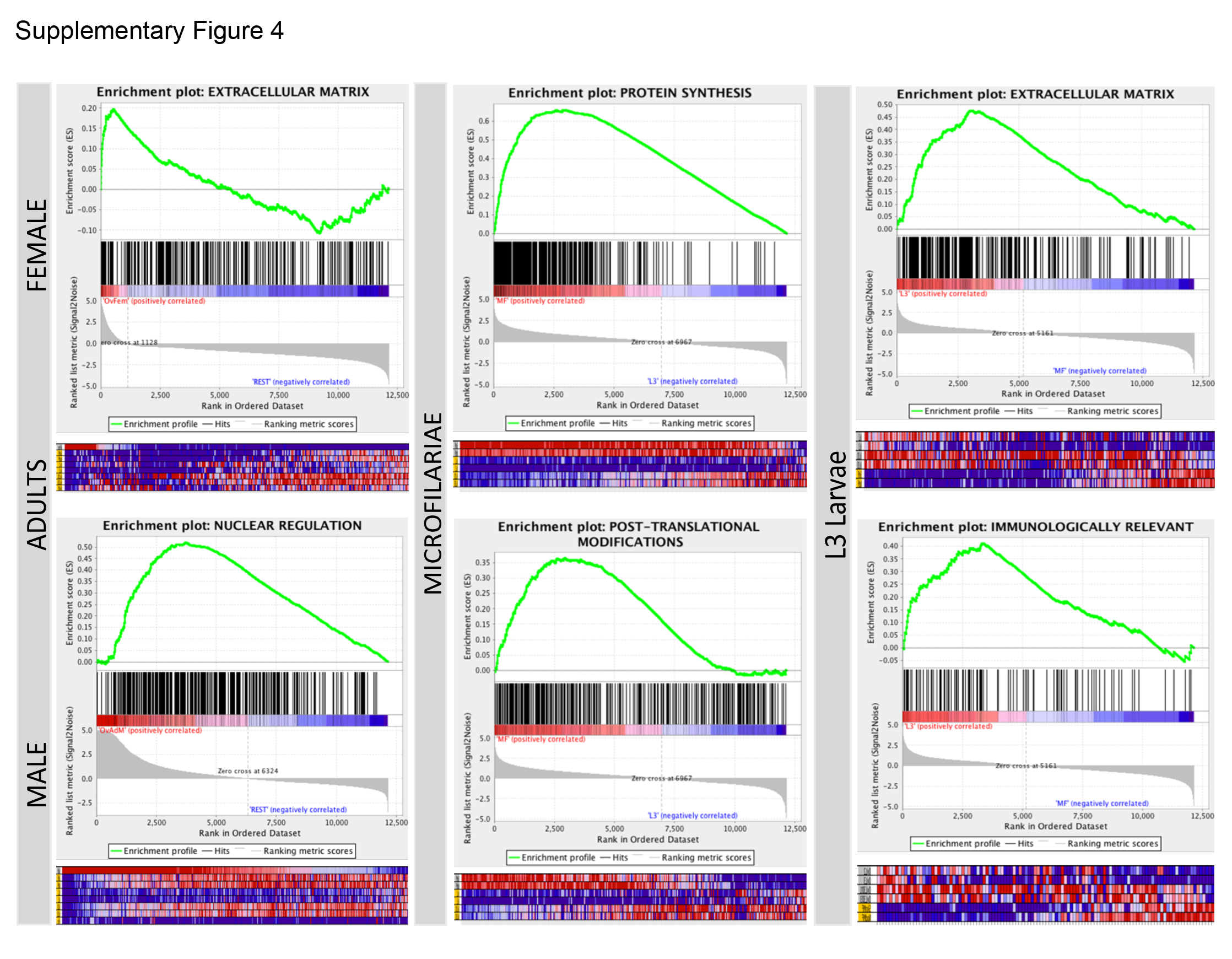

Supplement: Figure S4 — GSEA. Illustrated is the stage-specific enrichment of gene sets involved in specific functional categories in O. volvulus adults (left), in mf (middle), and during L3-L4 development (right). GSEA of transcripts ranked by their relative abundance in each stage was performed. The green curve shows the enrichment score and reflects the degree to which each protein (represented by the vertical lines) is represented at the top or bottom of the ranked list. The heat maps below the curves depict the relative abundances (red to blue indicates high to low expression) of the transcripts specifically enriched in a specific stage in comparison with other stages in the corresponding functional classes. Download [file mbo006163100sf4.tif]

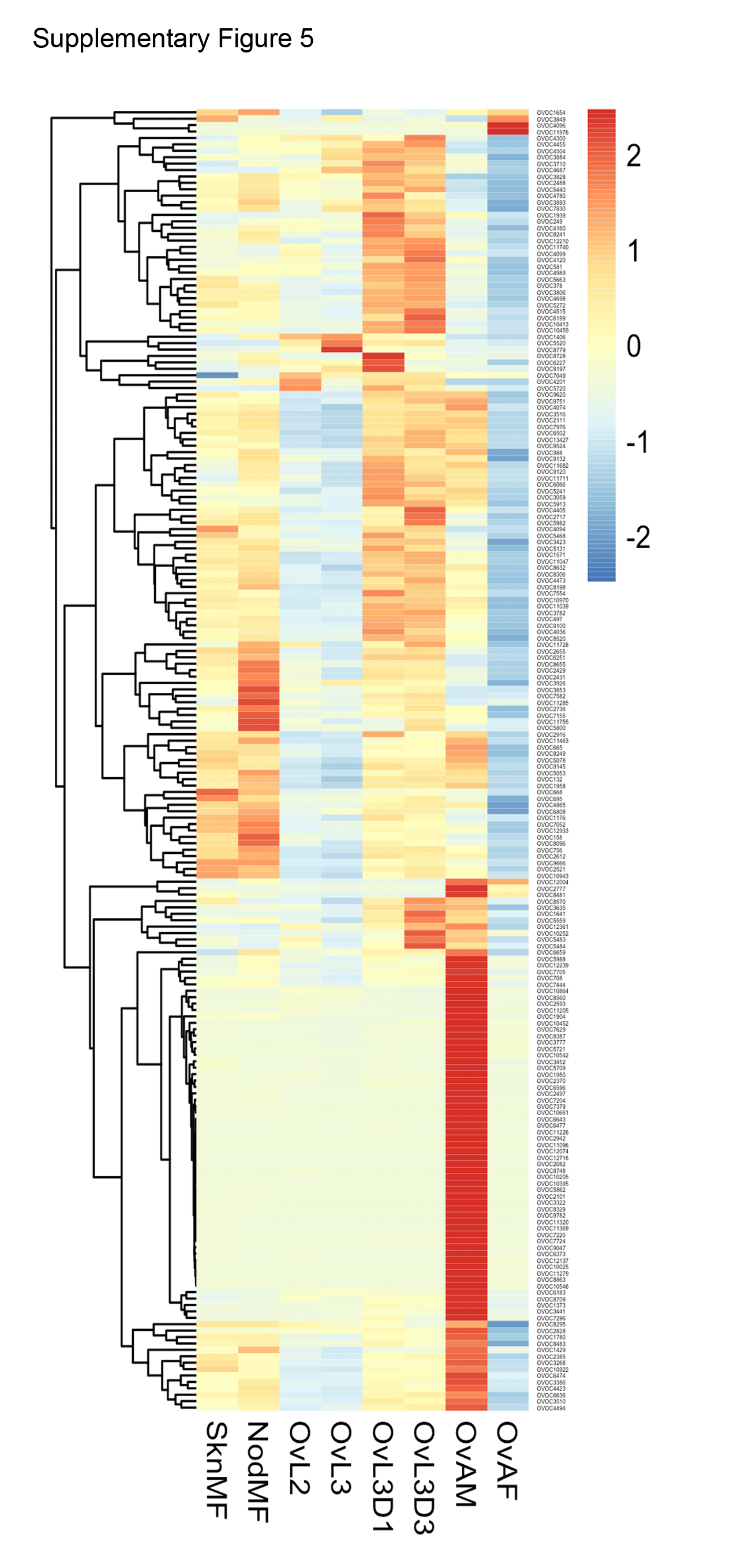

Supplement: Figure S5 — O. volvulus stage-specific expression of protein kinases. The heat map shown depicts the stage-specific expression (log2-transformed RPKM) of Pkinase (PF00069) domain-containing proteins. Red to blue denotes high to low expression, as shown on the right. Download [file mbo006163100sf5.tif]

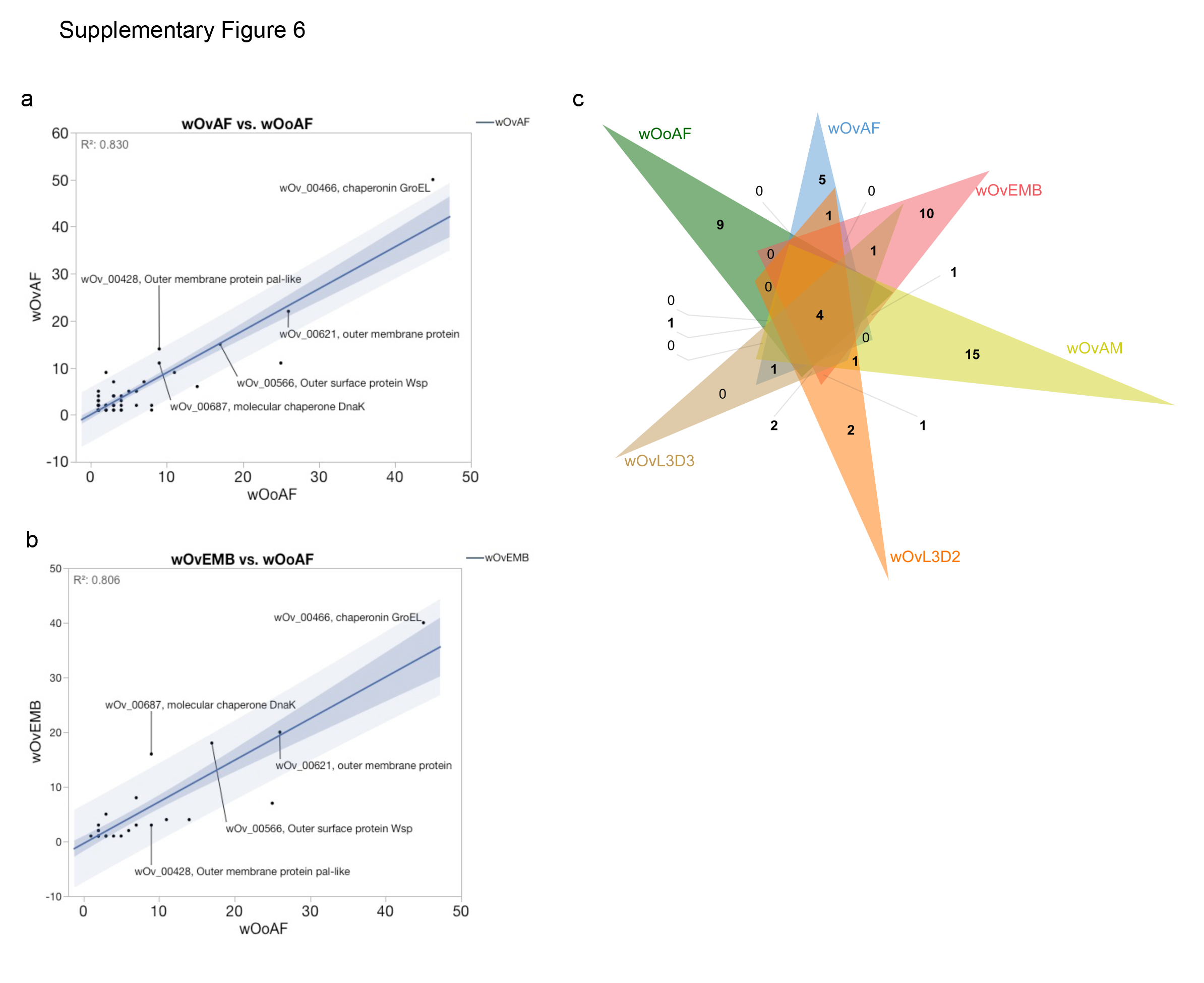

Supplement: Figure S6 — Comparative analysis of wOv proteins. (a) The correlation between the number of peptides identified in Wolbachia bacteria derived from adult females of O. volvulus (wOv; wOvAF) and in Wolbachia bacteria from adult females of O. ochengi (wOo; wOoAF). (b) The relationship between the numbers of peptides identified in Wolbachia bacteria derived from embryos of O. volvulus (wOv; wOvEMB) and in Wolbachia bacteria derived from adult females of O. ochengi (wOo; wOoAF). (c) The Venn diagram illustrates the four most abundant wOv proteins detected across all of the stages. Download [file mbo006163100sf6.tif]
